# Trinucleotide repeats and protein folding and disease: the perspective from studies with the androgen receptor

**DOI:** 10.4155/fso.15.47

**Published:** 2015-09-01

**Authors:** Folake A Orafidiya, Iain J McEwan

**Affiliations:** 1 School of Medical Sciences, Cell, Developmental & Cancer Biology Programme, University of Aberdeen, Foresterhill, Aberdeen, AB25 2ZD, Scotland, UK

## Abstract

The androgen receptor (AR), a ligand activated transcription factor plays a number of roles in reproduction, homeostasis and pathogenesis of disease. It has two major polymorphic sequences; a polyglutamine and a polyglycine repeat that determine the length of the protein and influence receptor folding, structure and function. Here, we review the role the folding of the AR plays in the pathogenesis of spinal-bulbar muscular atrophy (SBMA), a neuromuscular degenerative disease arising from expansion of the polyglutamine repeat. We discuss current management for SBMA patients and how research on AR structure function may lead to future drug treatments.

**Figure 1.**
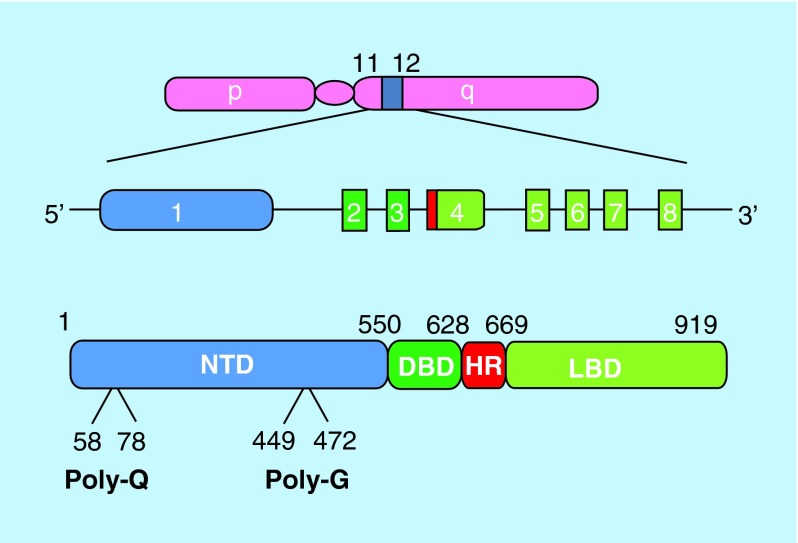
**Organization of the androgen receptor gene and protein.** The *Ar* gene is located on the long arm of the X-chromosome. The coding sequence consists of eight exons which give rise to the four structural and function domains of the receptor protein (exons and protein domains appear in the same colors). The location of the polymorphic amino acid repeats polyglutamine (Q21) and -glycine (G23) are indicated. DBD: DNA binding domain; HR: Hinge region; LBD: Ligand binding domain; NTD: N-terminal domain.

**Figure 2.**
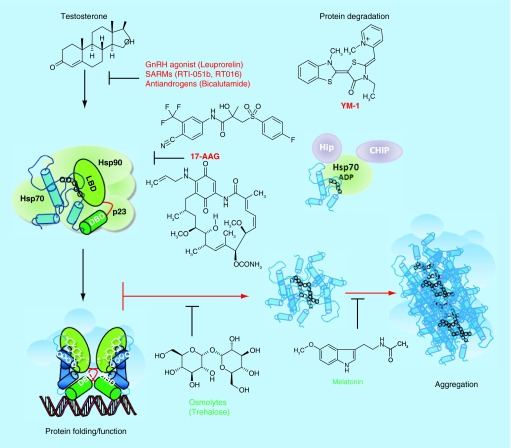
**Pathophysiological action of the AR protein and potential sites for therapeutic intervention in spinal-bulbar muscular atrophy.** Left Panel: in the absence of hormone the AR is located in the cytoplasm in a multiprotein complex) including molecular chaperones (hsp90, hsp70; green shapes). Upon binding hormone, the receptor translocates to the nucleus and binds as a dimer to specific DNA sequences and interacts with co-regulatory proteins and the transcription machinery (blue shapes). Right Panel; The AR-NTD is structurally flexible and the expanded AR poly-Q tract allows transition into a distinct conformation that may cause toxicity as a monomer or it may self-associate to form toxic oligomers, which could assemble into larger aggregates leading to intracellular inclusions. Formation of aggregates may also involve proteolysis and the generation of poly-Q containing N- terminal domain polypeptides. The principal toxic effects of the aberrantly folded protein may include alterations in transcription (through sequestering of co-regulatory proteins; blue shapes), metabolism or impairment of the proteasome or stress response pathways. Allosteric activators of Hsp70, including Hip and the small molecule YM-1 together with chaperone-dependent ubiquitin ligases, such as CHIP (green and purple shapes), increase substrate binding affinity, facilitate client protein ubiquitination and promote poly-Q AR clearance by the proteasome. Hormone binding and nuclear translocation can be prevented using GnRH agonists or possibly antiandrogens, while inhibiting Hsp90 (17-AAG) can block hormone binding and lead to degradation of the AR protein. Chemical chaperones, known as osmolytes may allow misfolded AR back into a native conformation, and thereby restore proper folding and functions of the receptor, while melatonin has been shown to reverse aggregate formation of the mutant receptor. See text for details and relevant references.

## The androgen receptor protein & domain structure

The androgen receptor (AR; NR3C4) is a 110 kDa protein and belongs to the ligand activated nuclear receptor superfamily. The AR is widely expressed in the body with high levels in the epididymis, prostate and skeletal muscles. The principal endogenous ligands of the AR are testosterone and its more potent analog dihydrotestosterone (DHT), both of which control phenotypic male sexual development, maintenance of bone mass and musculoskeletal development. Aside from its role in various physiological and homeostatic processes, the AR has also been directly implicated in a number of diseases such as hypogonadism, androgen insensitive syndrome (AIS), benign prostatic hyperplasia (BPH), initiation and progression of prostate cancer, spinal-bulbar muscular atrophy (SBMA), as well as male pattern baldness and osteoporosis [1].

The *AR* gene resides in the q11–12 portion of the long arm of the X-chromosomes (Figure 1); as only a single copy is found in males, the phenotypic expression of mutations is inevitable. Eight exons encode for the four distinct domains of the human AR protein, with varied lengths of polyglutamine and polyglycine repeats which account for the disparity in the exact amino acid number of the full-length protein (Figure 1). The domains include the hypervariable amino-terminal domain (NTD), the highly conserved DNA binding domain (DBD), the hinge region (HR) and the ligand binding domain (LBD) (Figure 1). The LBD is made up of a 12 α-helices forming a ligand binding pocket where hormone binds and a surface pocket for co-regulator protein interactions, termed activation function AF2. The AF2 pocket is also involved in interdomain interaction with an FQNLF motif of the NTD (N/C interaction) [2].

In the absence of hormone the receptor is predominantly cytoplasmic and found in a heterocomplex with molecular chaperones hsp90 and hsp70 and associated proteins which help maintain the receptor in a conformation with a high affinity for hormone (Figure 2). Once ligand binds, the conformation of the AR protein changes, which leads to nuclear translocation, DNA binding and downstream expression of androgen target genes, which in turn results in cell proliferation, differentiation, apoptosis and protein secretions depending on the target tissue [3].

The NTD constitutes about 60% of the AR protein and this region is structurally flexible and intrinsically disordered in the absence of binding partners [4]. Deletion of the LBD domain results in a constitutively active receptor with activity similar to that seen in full length ligand-bound receptor. This provides evidence for the transcriptional activity of the NTD. In contrast, deletion of part or the entire domain has been reported to result in weak transcriptional activity further denoting the importance of this region to the activity of the AR protein [5]. The region of the NTD termed the activation function 1 (AF1) mainly mediates this transactivation activity of the receptor [6].

### The receptor NTD & polymorphisms in polyglutamine & polyglycine repeats

Several amino acid repeat sequences are located in the NTD, with the two largest, a polyglutamine repeat (poly-Q) and a polyglycine repeat (Poly-G), being highly polymorphic (Figure 1). The poly-Q tract is coded by (CAG)_n_CAA and spans from 9 to 36 glutamine residues in the normal population [7–9]. There is an ethnicity difference in the length of CAG repeat which is shortest in Africans, medium in Caucasians and longest in Asians [10]. The high variability in the number of repeats may be as a result of DNA polymerase slippage during replication [11]. Longer poly-Q tracts tend to weaken AR activity and are associated with cryptorchidism, infertility and hypogonadism while shorter tract is associated with increased activation of the AR, which is linked to an increased risk of prostate cancer [12].

The triplets GGC/GGG/GGT code for the poly-G repeat which ranges from 10–27, with the predominant allele having 23 repeats [13]. Although not much has been reported on this polymorphism in isolation, tracts with length other than 23 are associated with lower transactivating ability [14].

The phenotypic expression of these polymorphisms varies in different ethnic groups. A number of population studies have shown a correlation between these polymorphisms and infertility; however, this is not true for all ethnic groups (Table 1).

These polymorphisms have also been shown to have an influence on bone mineral content and bone mineral density [15]. Longer tracts of CAG and GGN are reported to protect women against dyslipidemia and men form developing insulin resistance [16].

## SBMA: a protein misfolding disease?

AIS, cryptorchidism and prostate cancer are examples of diseases that result from point mutations in the receptor gene and have also been associated with varied repeat lengths of poly-Q and poly-G tracts in population studies (reviewed by [17]). However, in this review, our focus will be on the effect of poly-Q expansion and receptor protein misfolding in SBMA. As the function of the AR is mostly dependent on the NTD, changes in length or conformation of this domain are likely to have a significant impact on the receptor protein folding and function.

SBMA is an adult onset neuromuscular disease inherited via a recessive X-chromosome, therefore, it affects only males. It was first described in 1961 by Dr WR Kennedy, hence the name Kennedy's disease. SBMA affects about one in 50,000 males. It is reported in individuals with European or Asian background and it is more prevalent in the Japanese than any other ethnic group [18]. Affected persons usually have an expanded poly-Q tract between 38 and 62 residues [7–9]. Similar to other triplet repeat disorders, the length of the poly-Q expansion correlates inversely with age of onset, with disease severity and progression rate [19]. Lower motor neurons in the anterior horn of the spinal cord and those in the brainstem motor nuclei are predominantly affected in SBMA. Patients present with proximal limb muscle atrophy and weakness, fasciculation contractions, dysphagia, dysarthria, tremor, oilgospermia, gynecomastia and testicular atrophy [20,21].

Histopathologically, the distinctive feature of SBMA is the occurrence of nuclear inclusions (NI) in neural tissues of the brainstem and spinal cord as well as nonneural tissues such as the skin, prostate and testes. The accumulation of misfolded AR and aggregated mutant protein in the nuclei of motor neurons initiates neuronal dysfunction and eventual cell death. AR aggregates exist in two forms, an annular nonpathogenic oligomer with length of about 120–180 nm and a fibrillar toxic form with its length ranging from 300–600 nm. Expanded poly-Q AR induces fibrillar aggregation (Figure 2) [22]. However, the role of these inclusions in terms of disease initiation and/or progression remains less clear; although microaggregates of possibly proteolytic fragments of the AR may be the principal culprit in SBMA [7,9].

Changes in the content of the secondary structure and folding of the NTD are modulated by the length of the poly-Q repeat. Circular dichroism analysis of the AR-NTD revealed a dynamic picture of secondary structure content depending on the presence and length of the poly-Q repeat. The AR-NTD cloned from the genomic DNA of a patient with Kennedy's disease, with Q45, showed a modest increase in α-helix and decrease in β-structures [23]. Significantly, the hydrophobic helix stabilizing solvent TFE amplified the secondary structure properties of the AR-NTD with the expanded repeat. Interestingly, we also observed that deleting the repeat sequence decreased the α-helix content and the propensity of the AR-NTD to adopt helical conformations [23]. Crucially, these studies emphasized the importance of studying the structural properties of the poly-Q region in the context of the native protein. Earlier studies also found that disrupting the normal poly-Q repeat with leucine residues, effectively making shorter stretches of glutamines, resulted in a more stable conformation in the AR-NTD [24].

Structural analysis of the poly-Q repeat in the NTD of the Huntingtin protein (Htt), responsible for Huntington's disease, similarly demonstrate the plasticity in protein conformation and the importance of flanking sequences for the folding, misfolding and secondary structure content of the poly-Q repeat. For example, the C-terminal flanking polyproline sequence promoted a polyproline type II-like helix and inhibited β-sheet structure in the poly-Q repeat; leading to the conclusion that the flanking sequence influenced the ‘threshold’ at which aggregation could occur [25] while an ensemble of conformations (α-helix, random coil and extended loop) was observed for a poly-Q repeat length of 17 residues in crystal structures of the Htt NTD [26]. Dynamic modeling simulations also revealed that the polyproline stretch reduced the chance of the poly-Q repeat forming β-rich structure. Furthermore, it was observed that expansion of a poly-Q repeat resulted in a α-helix to β-sheet transition for the N-terminal flanking 17 amino acid sequence [27]. This region has also been reported to exist as disordered monomers and an α-helical tetramer and to be responsible for increased protein aggregation [28]. The presence of an α-helical coiled-coil structure has been reported for poly-Q proteins and correlated with both function (i.e., protein–protein interactions) and aggregation [29]. Collectively, the structural analysis of the AR-NTD and the Htt exon 1 polypeptides demonstrate that the poly-Q regions can exist as an ensemble of structures whose flexibility or stability is influenced by flanking sequence and cellular context (see below).

## Cellular context & AR pathophysiology

Based on extensive biochemical, cellular and animal studies it has been suggested that poly-Q repeat diseases share a similar mechanism of pathogenesis, which involves the expanded poly-Q tracts acquiring a nonnative conformation, which then aggregate and form inclusions. Aside from poly-Q expanded AR, inclusions contain chaperone and co-regulatory proteins and components of the ubiquitin–proteasome system which possibly attempt to refold or degrade mutant proteins (Figure 2) [30]. However, there is also evidence that aggregation maybe a protective mechanism of the cell and it has been proposed that there is also a toxic gain of function in mutant protein. Irrespective of the exact mechanism, it is clear that cellular context plays a part in pathogenesis.

As testosterone plays a critical role in the regulation of AR action, this hormone has been implicated in pathogenesis of SBMA. Chevalier-Larsen and colleagues observed that transgenic male mouse models of SBMA developed more severe motor dysfunction and presented with an earlier disease onset than females. Castrated symptomatic mice also showed an improved phenotype over time [31]. Heterozygous females are reported to be asymptomatic while homozygous females exhibit very mild symptoms [32] indicating that higher levels of circulating androgens in males may serve as a prerequisite in disease pathogenesis. This was confirmed when testosterone was administered to female transgenic mice, although they showed mild symptoms initially, continued administration of testosterone worsened the symptoms to levels comparable to male phenotypes [33]. Leuprorelin, a potent gonadotrophin releasing hormone receptor (GnRHR) agonist, was shown to rescue nuclear accumulation of mutant AR and motor dysfunction in transgenic mouse model. A reversal in behavioral and histopathological phenotypes was also seen in treated animals [34]. Put together, these studies postulate the role of androgens in disease development and progression (Figure 2). However, administration of testosterone to 2-month-old transgenic male mice for a period of 6 months failed to affect cellular markers of disease, histopathological and behavioral measures of disease [35]. This in essence shows that exogenous androgens do not exacerbate disease and that endogenous hormone levels are sufficient to saturate the AR for disease onset and progression.

The role of androgen in SBMA pathogenesis cannot be overemphasized but the detailed mechanisms leading to neurodegeneration remains unclear. Recent research has focused on various aspects of the AR native functions and metabolism – from its activation and translocation to the nucleus, to its interaction with co-regulatory proteins and regulation of target genes – and the role each step plays in misfolding, aggregation and toxicity. Interestingly, the combined use of the antiandrogen bicalutamide, to block nuclear translocation, and the disaccharide trehalose reduced aggregation of the AR with an expanded poly-Q [36]. Trehalose was shown to act by increasing autophagy, allowing for the degradation of the mutant receptor protein. We and our collaborators have found that this osmolyte can increase the secondary structure content of the polypeptide AR-NTDQ45 [khan sh, watt k, kumar r, mcewan ij, unpublished data]. Therefore, trehalose may act at different steps, including protein folding, to reduce AR toxicity (Figure 2).

As translocation of the AR to the nucleus is ligand dependent, androgens facilitate the translocation of poly-Q expanded AR and nuclear localization has been correlated with pathogenesis [37]. However, in a *Drosophila* model of SBMA, nuclear translocation of poly-Q expanded AR was not sufficient to initiate neurodegeneration. These findings implicate the importance of DNA binding by mutant AR as prerequisite to toxicity. In addition, it was also found that a functional AF2 domain and interaction with its coregulators were necessary for toxicity [38]. Thus, nuclear localization of poly-Q expanded AR is necessary, but not sufficient for nuclear aggregation and toxicity.

A major conformational change the AR undergoes after ligand activation is the interaction between the FQNLF motif of the NTD and the AF2 of the LBD (N/C interaction), which stabilizes the receptor facilitated hormone binding and recruitment of coregulators. Genetic mutation of this motif and the use of ligands, such as the antiandrogen bicalutamide and selective androgen receptor modulators (SARMS: RRI-05b, RT016) (Figure 2), that prevent the N/C interactions, identified this step as critical for the formation of mutant AR aggregate and toxicity [38,39].

Ligand dependent and independent post translational modifications of the AR have been shown to play a role in neurotoxicity. Phosphorylation of the AR has been shown to be dependent on poly-Q length, where an expanded repeat induces a conformational change that activates the MEK-1/2 pathway and enhances serine phosphorylation of mutant AR. Phosphorylation of serine-514 promotes cytotoxicity by augmenting caspase-3 cleavage of the AR creating amino-terminal fragments containing poly-Q repeats that are cytotoxic (Figure 2) [40]. By contrast, mutation of serines-215 and -792, which are Akt phosphorylation sites, to the phosphomimic residue aspartic acid was shown to prevent nuclear translocation, transactivation and toxicity associated with poly-Q expansion due to reduced ligand binding [41]. Similarly, treatment with IGF-1, which activates the PI3K/Akt pathway was found to reduce AR-induced toxicity [41]. Significantly, a recent study [42] has shown that methylation of the arginine residues, 210, 212, 787 and 789, by the enzyme protein methyltransferase 6 enhanced ARpolyQ toxicity. These arginine residues lie within the Akt phosphorylation consensus and Pennuto and co-workers propose a model where these posttranslational modifications, are mutually exclusive and, regulate hormone binding and neurodegeneration in models of SBMA [42].

The neurodegeneration observed in SBMA has been associated with increased activation of the JNK pathway by poly-Q expanded AR which inhibits binding of kinesin-1 to microtubules, which, in turn, disrupts fast axonal transport leading to neuronal dysfunction [43]. In a related study, the N-terminal fragments of mutant AR in SBMA were shown to activate the proapoptotic factors JNK and Bax that mediate neuronal apoptosis [44]. Another model for disease pathogenesis proposes the upregulation of calcitonin gene-related peptide α (CGRP1) by mutant AR which activates the JNK pathway enhancing damage in neuronal cells [45].

Acetylation of the AR has also been implicated in SBMA pathogenesis. Expanded poly-Q AR is hyperacetylated thus stabilizing the mutant AR and promoting nuclear aggregation. Pharmacologic reduction and mutations to inhibit acetylation at lysine 630, 632 and 633 significantly decreased aggregation and completely abolished toxicity in motor neurons. Furthermore, Montie and colleagues showed that SIRT1, a deacetylase of the AR protects against neurotoxicity by deacetylating expanded poly-Q AR [46]. Lysines 385 and 518 in the AR-NTD are also targets for the attachment of SUMO-1, resulting in sumolyation of the receptor. Upregulation of SUMO conjugates may antagonize the development of mutant protein aggregates thus serving as a neuroprotective mechanism [47]. In contrast, Lieberman and co-workers [48] observed that prevention of sumoylation enhanced receptor-dependent transcription and rescued SBMA-like phenotype in a mouse model.

Although the pathogenic mechanism of SBMA seems complex, and is likely to involve multiple steps, the above studies emphasize that an androgen-dependent activation of an expanded poly-Q AR is necessary for the neurodegeneration observed in SBMA and posttranslational modification of the receptor can enhance or ameliorate receptor toxicity.

## Current therapeutic interventions & disease management

As with other ‘protein-misfolding’ diseases, palliative care is offered to SBMA patients to minimize disability and improve their quality of life at each stage of disease as there is currently no effective pharmacologic therapy. Symptoms such as weakness and wasting of limb muscles are managed by the use of walkers, canes or braces. In later stages of disease, patients are confined to a wheel chair or other assistive device [49]. Patients presenting with bulbar weakness which can cause choking should be counseled on type and size of food they can eat and safest way to swallow: a feeding tube maybe used in advanced stages. Noninvasive ventilation aids may serve as tools to assist in breathing. Individuals with gynecomastia can undergo surgery to reduce breast size. Although overall quality of life is reduced in advanced stages, life expectancy of SBMA patients are not typically affected especially when life-threatening complications such as aspiration, asphyxiation and falling are properly managed [49].

As the knowledge of disease mechanism continues to increase, a number of therapeutic interventions to prevent or cure SBMA have been investigated. Androgens have been shown to play a significant role in pathogenesis, hence androgen ablation therapy could be an effective first-line target (Figure 2). Leuprorelin, which suppresses the secretion of gonadal sex hormones, has been used successfully in prostate cancer to lower the amount of circulating androgens. Thus, leuprorelin attenuated nuclear accumulation of mutant AR and motor dysfunction in SBMA transgenic mouse model [34]. As a result, Shimohata and colleagues carried out a 5-year follow-up on a SBMA patient with coexisting prostate cancer treated with this GnRH agonist. After a month, there was a rapid aggravation of gait disturbances. This could have resulted from the initial surge of androgens which is characteristic of GnRH receptor agonists [50]. Subsequently, there was an improvement in muscular weakness and long-term stabilization of motor function. Although, muscle weakness and atrophy had not disappeared completely as of the time of reporting, the authors proposed that leuprorelin is effective in advanced stages of SBMA [51].

Significantly, in a randomized, placebo-controlled Phase II clinical trial administration of leuprorelin to 50 recruited SBMA patients for 144 weeks, led to better motor functional scores and improved swallowing parameters were observed in treated patient as against those given placebo [52]. In addition, there was suppression of nuclear accumulation and stabilization of expanded poly-Q AR in motor neurons of the brainstem and spinal cord in autopsy specimens. Mutant AR accumulation in scrotal skin biopsy was also decreased. The study concluded that leuprorelin appears to inhibit functional deterioration in SBMA patients [52]. In a follow-up multicenter study, the efficacy and safety to leuprorelin was examined. Although well tolerated, leuprorelin was found not to exhibit significant effects on swallowing functions in SBMA patients [53]. Less successful was the trail involving the 5α-reductase inhibitor, dutasteride, which prevents the conversion of testosterone to DHT. No significant effect on the progression of muscle weakness over the 24-month period of investigation, was observed [54].

In as much as animal studies show convincing evidence for the use of androgen depleting compounds in SBMA therapy, clinical trials in humans has not establish efficacy. There is, therefore, a need to look beyond hormone therapy in the treatment and management of this slowly progressive condition in humans.

## Future research focus & its impact on development of novel therapies

Although the number of compounds going from preclinical to clinical trials are very limited, this has not deterred researchers investigating novel therapies to treat SBMA. One strategy has been to target the molecular chaperone machinery and in a recent paper Lieberman and co-workers elegantly demonstrated that regulation of the Hsp70 chaperone complex could reduce neurotoxicity and promote turnover of the AR. Of particular significance was the identification of a small molecule, YM-1, that allosterically activated Hsp70 (Figure 2) [55]. In other studies, inhibiting Hsp90 with the compound 17-AAG (17-(allylamino)-17-demethoxygeldanamycin), disrupted hormone binding and facilitated degradation of the mutant AR (Figure 2) [56]. Osmolytes are solutes which are abundant in living cells that act by interacting with the polypeptide backbone or by acting as a nucleus for hydration, which encourages protein folding and stability [57,58]. Kumar proposed that these ‘chemical chaperones’ can directly reverse the misfolded mutant AR back to its original conformation preventing aggregation and restoring proper function [57]. Interestingly, the osmolyte trehalose has already been reported to reduce aggregation of expanded poly-Q proteins and rescue the neurotoxic phenotype in a model of Huntington's disease [58] and a cell model of SBMA [36]. Furthermore, osmolytes have also been observed to modulate conformational changes within Hsp90 [59], which is part of the chaperone machinery complexed with the unliganded AR. Naturally occurring osmolytes have an added advantage in that they can easily cross the blood–brain barrier thus serve as a potential therapeutic tools [60].

Reversal of fibrillar aggregates to nontoxic annular ones can also have a therapeutic potential. Melatonin was shown to cause poly-Q expanded AR to form annular oligomers in neuronal cells in culture or *Drosophili*a motor neurons [22]. By contrast, compound B2 was shown to reduce mutant AR toxicity in SBMA cell and fly models by increasing accumulation of mutant AR into inclusions [61]. As formation of inclusion is thought to be a protective mechanism, compounds that promote aggregation into inclusion bodies may have therapeutic properties. Taken together, the above studies support the idea that compounds that target protein folding, degradation and aggregation may be viable therapies.

Decreasing the expression of poly-Q AR may be a feasible way of tackling SBMA. Adeno-associated virus vector mediated delivery of the microRNA, miR-196a was shown to ameliorate SBMA phenotypes in mouse models by inhibiting the expression of CELF2. In fibroblast samples obtained from patient with SBMA, this intervention was found to downregulate both AR and CELF2 mRNAs. A similar observation was made in thoracic spinal cord of subjects [62].

Another approach has been to target different kinase pathways that affect AR signaling. For example, Morfini and colleagues proposed the JNK pathway as a promising target [43]. The 5-HT1B/D receptor agonist nartriptan was shown to suppress neurotoxicity by downregulating the expression of CGRP1 which inactivates JNK pathways thereby ameliorating motor impairment in mouse models [45].

Activation of the PI3K/Akt signaling by IGF-1 was shown to protect motor neuron cells against toxicity of poly-Q AR [41]. Intraperitoneal administration of mecasermin rinfabate, a recombinant human IGF-1 and IGF-1-binding protein 3 at onset of disease in mouse models increased activation of Akt and reduced poly-Q aggregation in skeletal muscle. An improvement in motor performance, muscular pathology was observed suggesting receptor phosphorylation as a viable drug target [63]. Other posttranslational modifications, such as acetylation and sumolytion, could similarly serve as drug targets [46,48].

Genistein, an isoflavone found in soybean and also regarded as a broad spectrum tyrosine kinase inhibitor was shown to promote dissociation of AR from ARA70 by phosphorylating AR at serine-515. This induces protein degradation and inhibits neuronal accumulation of mutant AR. In transgenic mice models, there was an improvement in motor performances and muscle pathology upon long term oral treatment [64].

To date the most promising therapeutic approaches have targeted the ligand activation of the AR or the folding/aggregation and turnover of the mutant receptor. However, as androgen ablation therapy alone seems to be inefficacious in clinical trials, the treatment of SBMA may ultimately lie in the use of a combination therapy approach.

**Table 1.** **Population studies looking at poly-Q and/or poly-G repeat lengths and male infertility.**

**Ethnicity**	**Predominant allele implicated**	**Type of infertility**	**Ref.**
Chilean	CAG = 21	Idiopathic sertoli cell only syndrome	[13]
	GGN = 24	Cryptorchidism and spermatogenic failure	
Chinese	GGN <23	Defective spermatogenesis	[65]
	CAG = 24–25	>2.5 fold risk of severe oligospermia and azospermia	
English	CAG ≥23	Undermasculinization of male genitalia	[66]
Indian	GGN = 15–26	Does not relate to infertility	[67]
Italian	CAG = 22/GGC = 17	Asthenozoospermic cases	[68]
Nigerian	CAG = 14–28	Does not relate to infertility	[69]
	GGN = 17–24		
Swedish	GGN <23	Decreased semen volume	[70]
Japanese	CAG = 14–32	Oligozoospermia	[71]
Iranian	GGN = 24	Cryptorchidism	[72]

**Key terms**

**Androgen receptor:** The androgen receptor is an intracellular receptor protein that binds the hormones testosterone and DHT and functions as a ligand-activated transcription factor.

**Spinal-bulbar muscular atrophy:** SBMA or Kennedy's disease is a late-on-set, progressive neuromuscular degenerative disease. SBMA results from expansion of a polyglutamine repeat to more than 40 residues.

**Intrinsic disordered structure:** Intact proteins or protein domains maybe described as being intrinsically disordered. This refers to a lack of a single stable structure and where conformation is best described as an ensemble of confers which can be more or less folded.

**Osmolyte:** Osmolytes such as disaccharide, trehalose, and the polyalcohol trimethylamine N oxide are natural small molecules that have been shown to help proteins fold and adopt native structures.

**Executive summary**

Expansion of the large polyglutamine repeat in the amino-terminal domain of the AR is responsible for the neuromuscular degenerative disease spinal-bulbar muscular atrophy.
Hormone binding and nuclear translocation of the receptor have both been implicated in the pathology of the disease; and may be considered necessary but not sufficient for disease progression.
Novel drug treatments for spinal-bulbar muscular atrophy are currently focusing on hormone binding, protein folding and degradation and receptor posttranslational modifications as the means of preventing or reducing the appearance of nuclear inclusions that contain aggregates of the androgen receptor and co-regulatory and chaperone proteins.

## References

[1] Smith LB, Mitchell RT, McEwan IJ (2013). *Testosterone: from Basic Research to Clinical Applications*.

[2] He B, Kemppainen JA, Voegel JJ, Gronemeyer H, Wilson EM (1999). Activation function 2 in the human androgen receptor ligand binding domain mediates interdomain communication with the NH2-terminal domain. *J. Biol. Chem.*.

[3] Prescott J, Coetzee GA (2006). Molecular chaperones throughout the life cycle of the androgen receptor. *Cancer Lett.*.

[4] Kumar R MI (2012). Allosteric modulators of steroid hormone receptors: structural dynamics and gene regulation. *Endocr. Rev.*.

[5] Farla P, Hersmus R, Geverts B (2004). The androgen receptor ligand-binding domain stabilizes DNA binding in living cells. *J. Struct. Biol.*.

[6] McEwan IJ (2012). Intrinsic disorder in the androgen receptor: identification, characterisation and drugability. *Mol. Biosyst.*.

[7] Jordan CL, Lieberman AP (2008). Spinal and bulbar muscular atrophy: a motoneuron or muscle disease?. *Curr. Opin. Pharmacol.*.

[8] La Spada AR, Wilson EM, Lubahn DB, Harding AE, Fischbeck KH (1991). Androgen receptor gene mutations in X-linked spinal and bulbar muscular atrophy. *Nature*.

[9] Fumiaki T, Masahisa K, Haruhiko B, Keisuke S, Hiroaki A, Gen S (2012). Current status of treatment of spinal and bulbar muscular atrophy. *Neural Plasticity*.

[10] Ackerman CM, Lowe LP, Lee H (2012). Ethnic variation in allele distribution of the androgen receptor (AR) (CAG)n repeat. *J. Androl.*.

[11] Gelmann EP (2002). Molecular biology of the androgen receptor. *J. Clin. Oncol.*.

[12] Palazzolo I, Gliozzi A, Rusmini P (2008). The role of the polyglutamine tract in androgen receptor. *J. Steroid Biochem. Mol. Biol.*.

[13] Castro-Nallar E, Bacallao K, Parada-Bustamante A (2010). Androgen receptor gene CAG and GGN repeat polymorphisms in chilean men with primary severe spermatogenic failure. *J. Androl.*.

[14] Lundin KB, Giwercman A, Dizeyi N, Giwercman YL (2007). Functional *in vitro* characterisation of the androgen receptor GGN polymorphism. *Mol. Cell. Endocrinol.*.

[15] Guadalupe-Grau A, Rodríguez-González FG, Ponce-González JG (2010). Bone mass and the CAG and GGN androgen receptor polymorphisms in young men. *PLoS ONE*.

[16] Rodríguez-González G, Ramírez-Moreno R, Pérez P (2009). The GGN and CAG repeat polymorphisms in the exon-1 of the androgen receptor gene are, respectively, associated with insulin resistance in men and with dyslipidemia in women. *J. Steroid Biochem. Mol. Biol.*.

[17] Rajender S, Singh L, Thangaraj K (2007). Phenotypic heterogeneity of mutations in androgen receptor gene. *Asian J. Androl.*.

[18] La Spada A, Pagan RA, Adam MP, Adringer HH (1999). Spinal and bulbar muscular atrophy. *GeneReviews™*.

[19] Igarashi S, Tanno Y, Onodera O (1992). Strong correlation between the number of CAG repeats in androgen receptor genes and the clinical onset of features of spinal and bulbar muscular atrophy. *Neurology*.

[20] Rhodes LE, Freeman BK, Auh S (2009). Clinical features of spinal and bulbar muscular atrophy. *Brain*.

[21] Dejager S, Bry-Gauillard H, Bruckert E (2002). A comprehensive endocrine description of Kennedy's disease revealing androgen insensitivity linked to CAG repeat length. *J. Clin. Endocrinol. Metabol.*.

[22] Jochum T, Ritz ME, Schuster C (2012). Toxic and non-toxic aggregates from the SBMA and normal forms of androgen receptor have distinct oligomeric structures. *Biochim. Biophy. Acta (BBA)*.

[23] Davies P, Watt K, Kelly SM, Clark C, Price NC, McEwan IJ (2008). Consequences of poly-glutamine repeat length for the conformation and folding of the androgen receptor amino-terminal domain. *J. Mol. Endocrinol.*.

[24] Buchanan G, Yang M, Cheong A (2004). Structural and functional consequences of glutamine tract variation in the androgen receptor. *Hum. Mol. Genet.*.

[25] Darnell G, Orgel JPRO, Pahl R, Meredith SC (2007). Flanking polyproline sequences inhibit β-sheet structure in polyglutamine segments by inducing PPII-like helix structure. *J. Mol. Biol.*.

[26] Kim MW, Chelliah Y, Kim SW, Otwinowski Z, Bezprozvanny I (2009). Secondary structure of Huntingtin amino-terminal region. *Structure*.

[27] Lakhani VV, Ding F, Dokholyan NV (2010). Polyglutamine induced misfolding of huntingtin exon1 is modulated by the flanking sequences. *PLoS Comput. Biol.*.

[28] Jayaraman M, Mishra R, Kodali R (2012). Kinetically competing Huntingtin aggregation pathways control amyloid polymorphism and properties. *Biochemistry*.

[29] Fiumara F, Fioriti L, Kandel ER, Hendrickson WA (2010). Essential role of coiled coils for aggregation and activity of Q/N-rich prions and PolyQ proteins. *Cell*.

[30] Adachi H, Waza M, Tokui K (2007). CHIP overexpression reduces mutant androgen receptor protein and ameliorates phenotypes of the spinal and bulbar muscular atrophy transgenic mouse model. *J. Neurosci.*.

[31] Chevalier-Larsen ES, O'Brien CJ, Wang H (2004). Castration restores function and neurofilament alterations of aged symptomatic males in a transgenic mouse model of spinal and bulbar muscular atrophy. *J. Neurosci.*.

[32] Schmidt BJ, Greenberg CR, Allingham-Hawkins DJ, Spriggs EL (2002). Expression of X-linked bulbospinal muscular atrophy (Kennedy disease) in two homozygous women. *Neurology*.

[33] Katsuno M, Adachi H, Kume A (2002). Testosterone reduction prevents phenotypic expression in a transgenic mouse model of spinal and bulbar muscular atrophy. *Neuron*.

[34] Katsuno M, Adachi H, Doyu M (2003). Leuprorelin rescues polyglutamine-dependent phenotypes in a transgenic mouse model of spinal and bulbar muscular atrophy. *Nat. Med.*.

[35] Chevalier-Larsen ES, Merry DE (2012). Testosterone treatment fails to accelerate disease in a transgenic mouse model of spinal and bulbar muscular atrophy. *Dis. Model. Mech.*.

[36] Giorgetti E, Rusmini P, Crippa V (2015). Synergic prodegradative activity of bicalutamide and trehalose on the mutant androgen receptor responsible for spinal and bulbar muscular atrophy. *Hum. Mol. Genet.*.

[37] Takeyama K, Ito S, Yamamoto A (2002). Androgen-dependent neurodegeneration by polyglutamine-expanded human androgen receptor in drosophila. *Neuron*.

[38] Nedelsky NB, Pennuto M, Smith RB (2010). Native functions of the androgen receptor are essential to pathogenesis in a drosophila model of spinobulbar muscular atrophy. *Neuron*.

[39] Orr CR, Montie HL, Liu Y (2010). An interdomain interaction of the androgen receptor is required for its aggregation and toxicity in spinal and bulbar muscular atrophy. *J. Biol. Chem.*.

[40] LaFevre-Bernt MA, Ellerby LM (2003). Kennedy's disease: phosphorylation of the polyglutamine-expanded form of androgen receptor regulates its cleavage by caspase-3 and enhances cell death. *J. Biol. Chem.*.

[41] Palazzolo I, Burnett BG, Young JE (2007). Akt blocks ligand binding and protects against expanded polyglutamine androgen receptor toxicity. *Hum. Mol. Genet.*.

[42] Scaramuzzino C, Casci I, Parodi S (2015). Protein arginine methyltransferase 6 enhances polyglutamine-expanded androgen receptor function and toxicity in spinal and bulbar muscular atrophy. *Neuron*.

[43] Morfini G, Pigino G, Szebenyi G, You Y, Pollema S, Brady ST (2006). JNK mediates pathogenic effects of polyglutamine-expanded androgen receptor on fast axonal transport. *Nat. Neurosci.*.

[44] Young JE, Garden GA, Martinez RA (2009). Polyglutamine-expanded androgen receptor truncation fragments activate a bax-dependent apoptotic cascade mediated by DP5/Hrk. *J. Neurosci.*.

[45] Minamiyama M, Katsuno M, Adachi H (2012). Naratriptan mitigates CGRP1-associated motor neuron degeneration caused by an expanded polyglutamine repeat tract. *Nat. Med.*.

[46] Montie HL, Pestell RG, Merry DE (2011). SIRT1 Modulates aggregation and toxicity through deacetylation of the androgen receptor in cell models of SBMA. *J. Neurosci.*.

[47] Mukherjee S, Thomas M, Dadgar N, Lieberman AP, Iñiguez-Lluhí JA (2009). Small ubiquitin-like modifier (SUMO) modification of the androgen receptor attenuates polyglutamine-mediated aggregation. *J. Biol. Chem.*.

[48] Chua JP, Reddy SL, Yu Z (2015). Disrupting SUMOylation enhances transcriptional function and ameliorates polyglutamine androgen receptor-mediated disease. *J. Clin. Invest.*.

[49] Finsterer J (2010). Perspectives of Kennedy's disease. *J. Neurol. Sci.*.

[50] Cook T, Sheridan WP (2000). Development of GnRH antagonists for prostate cancer: new approaches to treatment. *Oncologist*.

[51] Shimohata T, Kimura T, Nishizawa M, Onodera O, Tsuji S (2004). Five year follow up of a patient with spinal and bulbar muscular atrophy treated with leuprorelin. *J. Neurol. Neurosurg. Psychiatry*.

[52] Banno H, Katsuno M, Suzuki K (2009). Phase 2 trial of leuprorelin in patients with spinal and bulbar muscular atrophy. *Ann. Neurol.*.

[53] Katsuno M, Banno H, Suzuki K (2010). Efficacy and safety of leuprorelin in patients with spinal and bulbar muscular atrophy (JASMITT study): a multicentre, randomised, double-blind, placebo-controlled trial. *Lancet Neurol.*.

[54] Fernández-Rhodes LE, Kokkinis AD, White MJ (2011). Efficacy and safety of dutasteride in patients with spinal and bulbar muscular atrophy: a randomised placebo-controlled trial. *Lancet Neurol.*.

[55] Wang AM, Miyata Y, Klinedinst S (2013). Activation of Hsp70 reduces neurotoxicity by promoting polyglutamine protein degradation. *Nat. Chem. Biol.*.

[56] Rusmini P, Simonini F, Crippa V (2011). 17-AAG increases autophagic removal of mutant androgen receptor in spinal and bulbar muscular atrophy. *Neurobiol. Dis.*.

[57] Khan SH, Ahmad N, Ahmad F, Kumar R (2010). Naturally occurring organic osmolytes: from cell physiology to disease prevention. *IUBMB Life*.

[58] Tanaka M, Machida Y, Niu S (2004). Trehalose alleviates polyglutamine-mediated pathology in a mouse model of Huntington disease. *Nat. Med.*.

[59] Street TO, Krukenberg KA, Rosgen J, Bolen DW, Agard DA (2010). Osmolyte-induced conformational changes in the Hsp90 molecular chaperone. *Protein Sci.*.

[60] Kumar R (2012). Role of androgen receptor polyQ chain elongation in Kennedy's disease and use of natural osmolytes as potential therapeutic targets. *IUBMB Life*.

[61] Palazzolo I, Nedelsky NB, Askew CE (2010). B2 attenuates polyglutamine-expanded androgen receptor toxicity in cell and fly models of spinal and bulbar muscular atrophy. *J. Neurosci. Res.*.

[62] Miyazaki Y, Adachi H, Katsuno M (2012). Viral delivery of miR-196a ameliorates the SBMA phenotype via the silencing of CELF2. *Nat. Med. Lett.*.

[63] Rinaldi C, Bott LC, Chen KL (2012). Insulinlike growth factor (IGF)-1 administration ameliorates disease manifestations in a mouse model of spinal and bulbar muscular atrophy. *Mol. Med.*.

[64] Qiang Q, Adachi H, Huang Z (2013). Genistein, a natural product derived from soybeans, ameliorates polyglutamine-mediated motor neuron disease. *J. Neurochem.*.

[65] Han TT, Ran J, Ding XP (2013). Cytogenetic and molecular analysis of infertile Chinese men: karyotypic abnormalities, Y-chromosome microdeletions, and CAG and GGN repeat polymorphisms in the androgen receptor gene. *Genet. Mol. Res.*.

[66] Lim HN, Chen H, McBride S (2000). Longer polyglutamine tracts in the androgen receptor are associated with moderate to severe undermasculinized genitalia in XY males. *Hum. Mol. Genet.*.

[67] Rajender S, Rajani V, Gupta NJ, Chakravarty B, Singh L, Thangaraj K (2006). No association of androgen receptor GGN repeat length polymorphism with infertility in Indian men. *J. Androl.*.

[68] Delli Muti N, Agarwal A, Buldreghini E (2013). Have androgen receptor gene CAG and GGC repeat polymorphisms an effect on sperm motility in infertile men?. *Andrologia*.

[69] Akinloye O, Gromoll J, Nieschlag E, Simoni M (2009). Androgen receptor gene CAG and GGN polymorphisms in infertile Nigerian men. *J. Endocrinol. Invest.*.

[70] Lundin KB, Giwercman YL, Rylander L, Hagmar L, Giwercman A (2006). Androgen receptor gene GGN repeat length and reproductive characteristics in young Swedish men. *Eur. J. Endocrinol.*.

[71] Komori S, Kasumi H, Kanazawa R (1999). CAG repeat length in the androgen receptor gene of infertile Japanese males with oligozoospermia. *Mol. Hum. Reprod.*.

[72] Radpour R, Rezaee M, Tavasoly A, Solati S, Saleki A (2007). Association of long polyglycine tracts (GGN Repeats) in Exon 1 of the androgen receptor gene with cryptorchidism and penile hypospadias in iranian patients. *J. Androl.*.

